# Patient and professional use of the root coverage esthetic score (RES) and how it relates to patient satisfaction following periodontal plastic surgery

**DOI:** 10.1186/s12903-022-02326-8

**Published:** 2022-07-18

**Authors:** Anne Judith Sørdahl, Anders Verket

**Affiliations:** Department of Periodontology, Faculty of Dentistry, Institute of Clinical Dentistry, University of Oslo, Geitmyrsveien 69, POB 1109, 0455 Oslo, Blindern, Norway

**Keywords:** Root coverage esthetic score, Patient satisfaction, Periodontal plastic surgery, Patient-reported outcomes

## Abstract

**Background:**

Following periodontal plastic surgery in the treatment of recession defects, previous studies have reported that patients rate the esthetic outcomes more favorable than dental professionals. The root coverage esthetic score has been developed and suggested to serve as a comprehensive assessment instrument as it addresses several esthetic outcomes following root coverage procedures. However, no study has yet reported on patient use of this instrument. In the present study clinical, esthetic and patient-reported outcomes following periodontal plastic surgery were assessed. The primary objective was to compare the esthetic/clinical outcome as judged by the patient and by one dentist by using the root coverage esthetic score. The secondary objective was to evaluate the correlation between patient-reported outcomes, root coverage esthetic score and clinical parameters following treatment of recession defects.

**Materials and methods:**

Subjects that had undergone periodontal plastic surgery were invited to score the treatment outcome according to the root coverage esthetic score, which subsequently also was professionally scored by a dentist. Thereafter, the subjects answered a questionnaire on patient satisfaction. All types of surgical root coverage procedures in canine or incisor teeth were included.

**Results:**

A total of 34 subjects were included, presenting 46 treated recessions. No statistically significant different score was found comparing the root coverage esthetic score by the patient and the professional. The majority of subjects was satisfied with the treatment outcome, and most would have undergone the treatment again.

**Conclusion:**

The root coverage esthetic score assessment can be conducted by patients and was not statistically significant different to that of the professional. Patient satisfaction is not always dependent on complete root coverage or the other clinical parameters included in the root coverage esthetic score.

## Background

The term mucogingival surgery was introduced in 1957 by Friedman and was defined as “surgical procedures designed to preserve gingiva, remove aberrant frenulum or muscle attachments, and increase the depth of the vestibule” [1]. In 1993 Miller proposed the term “periodontal plastic surgery” (PPS), which included correction of alveolar ridge deformities and soft tissue esthetics. However, this was redefined in 2014, where the aim of PPS was to modify the position of the gingival margin and/or the amount and characteristics of marginal soft tissues, at teeth or dental implants [2]. Mucogingival deformities or recessions may be congenital, developmental, or acquired defects. Gingival recession defects is defined as the apical shift of the gingival margin with respect to the cemento-enamel junction (CEJ) and is a common condition [3–7]. Previously, the most used classification system for gingival recession defects is the Miller classification [8]. A new classification system for gingival recession defects was introduced in 2011 [9]. Three recession types (RT) were identified; RT1 included gingival recession defects with no loss of interproximal attachment, RT2 included interproximal attachment loss less than or equal to the buccal site, and RT3 higher interproximal attachment loss than the buccal site.

Exposure of the root surface may lead to dentin hypersensitivity [4], esthetic concerns for the patient and caries/non-caries cervical lesions. There is also some evidence that untreated gingival recession defects in individuals with good oral hygiene may progress during long-term follow up [10, 11].

To date, most outcomes reported in studies of PPS concerns esthetic improvement, whereas data on alleviating clinical symptoms is limited. Dentin hypersensitivity is of concern for patients and may affect quality of life [12]. However, the evidence for an ameliorating effect of surgical RC is still scarce [13]. Perception of buccal recessions by patients and request for treatment was analysed in a recent study [7]. In this study the number of observed recessions by clinicians was large, but only a few of them were perceived by the patients and requested for treatment.

In a recent review it was concluded that few studies have evaluated patient satisfaction in a standardized approach following RC procedures, and that patient-reported evaluation is an important component of PPS [14]. Professional assessment to evaluate the treatment outcome, as well as patient-reported satisfaction, are both useful to assess PPS outcomes. A standardized scoring system for esthetic outcomes (RES), was introduced in 2009 [15], which may serve as a facilitated and standardized tool for clinicians to assess the treatment outcome. Complete success by objective measures requires a re-established gingival margin at the CEJ [16].

There are numerous studies in the literature on the treatment of gingival recession defects and several comparing the esthetic outcome after different treatment approaches [14, 17, 18]. A visual analog scale or a 5-point Likert scale is the tool most commonly used to determine patients’ satisfaction with the esthetic outcome [14, 17]. In a previous study it was demonstrated that patients appear to rate the esthetic results more favorable than professionals [19]. In the study, the clinicians seemed to consider the percentage of root-coverage to be essential, but this did not apply to the same extent for the patients.

To the best knowledge of the authors, no study has to date used the RES-system for evaluation by the patient. It is of interest to assess whether professional use of this scoring system is reflected by the opinion of the patients being assessed. The purpose of the present study was to evaluate the clinical, esthetic and PROs of patients who had undergone RC procedures.

The primary objective was to compare esthetic and clinical outcomes as judged by the patient and by a dentist professional by using RES. The secondary objectives were to explore the correlation between PRO, RES and clinical parameters following PPS of recession defects.

## Materials and methods

The Regional Ethics Committee approved the study (178985/2020). A search in the electronic journal system at the Faculty of Dentistry, University of Oslo, was performed to identify subjects who had undergone RC procedures since 2015 at the Department of Periodontology. Subjects were invited to a clinical examination if the treatment had been performed at least 6 months in advance.

Data collection took place between December 2020 and June 2021. Single or multiple buccal recession defects in mandibular or maxillary canine or incisor teeth were included. All types of surgical RC procedures were included.

Inclusion criteria were: (1) patients who had undergone RC procedures for recession defects in the period from 2015 to 2021, (2) > 18 years at the time of surgery, (3) single or multiple recession defects (RT1, RT2, or RT3) on mandibular or maxillary canine or incisor teeth, and (4) photographs and x-ray taken before the surgery.

### Patient-reported data

Patients were explained how to use the RES-system tool with the use of photos illustrating the various parameters. This was done individually with no time limit and included the five RES-factors: the level of the gingival margin (GM), marginal tissue contour (MTC), soft tissue texture (STT), mucogingival junction alignment (MGJ), and gingival color (GC) [15]. In the original system 0, 3 or 6 points are used for the evaluation of the position of the GM, whereas a score of 0 or 1 point is used for each of the other variables. In addition to the use of the original RES, we modified the scoring system for the GM-variable. For the evaluation of the position of the GM, the patient could score from 0 to 6 points. This modified RES (mRES) variable thus enabled five potential outcomes other than 0% (0 points) or 100% (6 points) RC. A pre-operative photograph of the relevant area was given to the patient along with a mirror for self-assessment.

Following RES-assessment by the patient, a two-part questionnaire was answered. The questionnaire included questions from previous studies reporting PROs [17, 19–22]. In the first part the patient filled out and addressed the indication for treatment, the likely etiology for the gingival recession defects and if they had undergone orthodontic treatment earlier in life. The second part of the questionnaire consisted of ten questions which addressed patient satisfaction, tooth hypersensitivity, esthetic outcomes and morbidity of the treatment, whether it was easier to clean the relevant area following treatment, if they would recommend the treatment to others and if they would do the same treatment procedure again, by use of a five-point Likert scale (1: poor; 2: fair; 3: neutral; 4: good; 5: excellent/1: strongly disagree, 2: disagree, 3: neutral, 4: agree, 5: strongly agree). They were also asked if they had orthodontic treatment in conjunction with and prior to the PPS. The patient completed this form prior to clinical measurements and professional RES and mRES evaluation.

### Professional assessment

An experienced dentist and postgraduate student in periodontology (AJS) conducted the RES and mRES assessments independently, without knowing the patient score. None of the recessions included were surgically treated by the examiner to avoid treatment outcome bias. The examiner was calibrated with an experienced periodontist (AV) before start to standardize the assessment. This was conducted by comparing pre- and postoperative photos of 15 treated recessions, and interrater reliability was calculated. At least one month following the clinical examination, the examiner (AJS) repeated the RES evaluation by photographs and compared it to that of the clinical RES assessment to determine intra-rater reliability.

Information on gender, age at the intervention, history of periodontitis, history of orthodontics, included tooth/teeth, recession type at baseline, periodontal pocket depth (PPD) at baseline and number of months postoperatively were collected. The examiner (AJS) recorded the clinical measurements by using a periodontal probe (LM 52B Si, ErgoNorm) and visual inspection. The following clinical measurements were obtained from the surgically treated tooth and adjacent teeth: periodontal probing depth (PPD), clinical attachment level (CAL), bleeding on probing (BoP), dichotomous plaque index (PI) [23], gingival recession defect depth, all at six sites per tooth. In addition, full-mouth plaque score (FMPS) was recorded, tooth mobility (I–III) [24], and the width of the keratinized buccal tissue was recorded at three sites per tooth. The total number of PPS of the treated tooth/teeth were also registered.

### Data analysis

Demographic data, information about the RC treatment and patient satisfaction was presented with descriptive statistics. RES data was examined for normality using Shapiro–Wilk normality test; and did not show normal distribution. Intra/inter-class correlation coefficient (ICC): two-way random mixed effects; consistency; absolute agreement, was used to compare RES assessments. For patient and professional RES and mRES comparisons, ICC was performed for each score (GM, MTC, STT, GC, MGJ). The interclass correlation coefficient between the dentists (AV and AJS) was 0.992 (CI 0.986–0.995) after calibration, reflecting high agreement. The intraclass correlation coefficient between the two examiner evaluations was 0.987 (CI 0.978–0.992).

For analysis of potential correlations between PRO (1–5), RES and RC, a Spearman rank order correlation was calculated. For comparison between treatment modalities an Anova on ranks was calculated. Analyses were performed using SPSS software (SPSS version 24, IBM, Armonk, NY, USA).

## Results

### Clinical outcomes

**Fig. 1:**
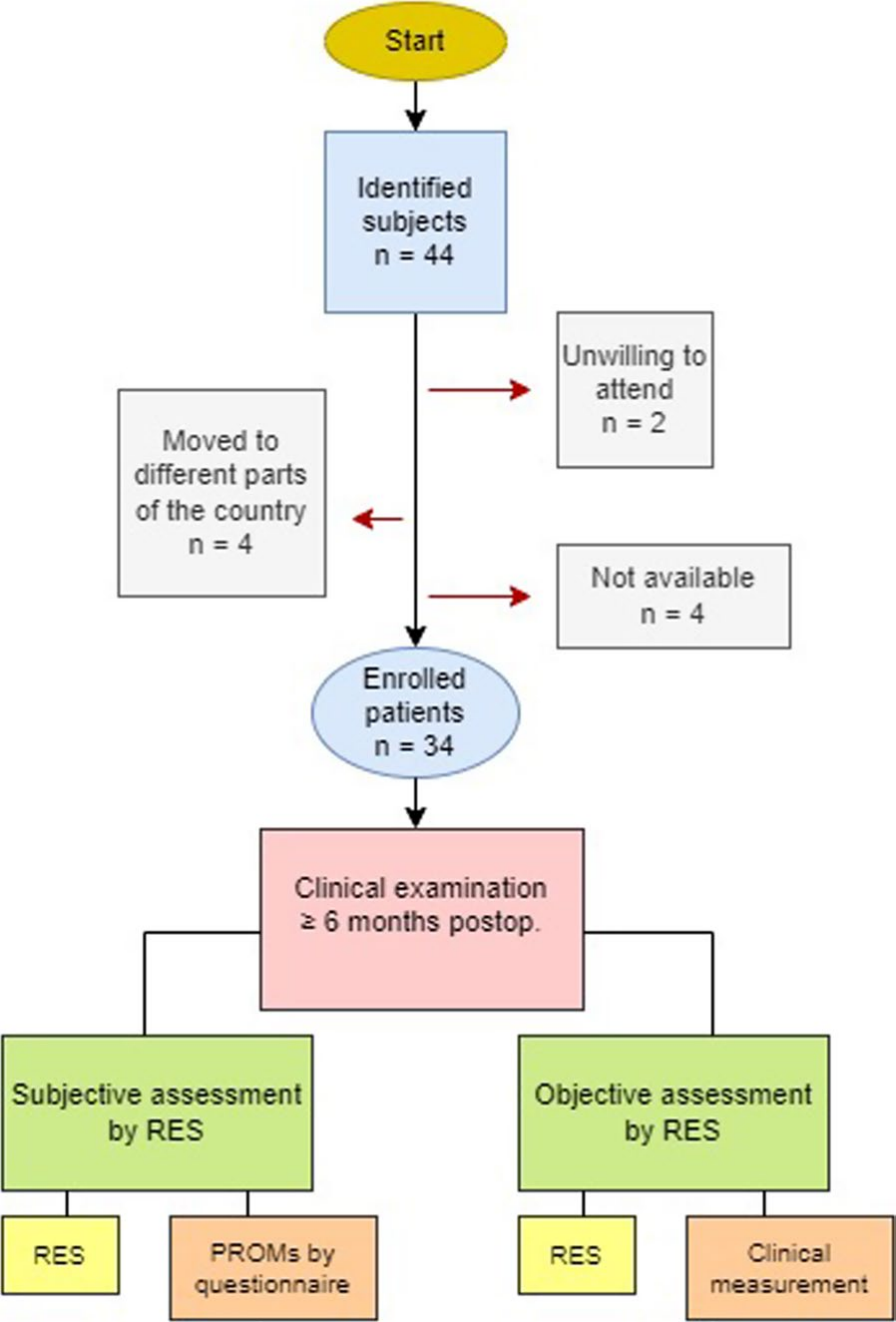
Flow chart

In the recruitment, 44 eligible patients were identified, of which 34 (77.3%) agreed to and attended a clinical examination. 24 females and 10 males with a mean age of 35.1 ± 11.8 (20–58) years were included. A detailed flow chart diagram of patient recruitment is shown in Fig. 1. The clinical examination was performed on average 20.7 months ± 13.6 following surgery (range 6–53 months).

Clinical parameters of the treated sites are provided in Table 1. Two patients had a history of periodontitis but demonstrated periodontal health [25]. Of the 46 treated recessions, 13 (28.3%) were maxillary and 33 (71.7%) mandibular, and a total of 29 recessions were single. RT at baseline consisted of 20 (43.5%) RT1, 24 (52.2%) RT2 and 2 (4.3%) RT3. All patients were non-smokers. Mean recession following treatment was 1.2 mm for RT1, 2.5 mm for RT2 and 3 mm for RT3. Different treatment approaches were used in the surgical treatment including coronally advanced flap (CAF) with or without connective tissue graft (CTG) (*n* = 31) [26], tunneling technique (TUN + CTG) (*n* = 8) [27], or vestibuloplasty surgery (VES ± CTG) (*n* = 7). Six recessions in 6 patients were surgically treated twice, and the second surgeries all included CAF with or without CTG.

**Table 1:** Clinical parameters for treated sites at baseline and examination

Mean	RT1	RT2	RT3	*p*-value
*Baseline data*				
Female (rec)	13 (17)	11 (13)	1 (2)	
Male (rec)	3 (6)	7 (8)		
Mean age (Range)	32 ± 8.61 [20–50]	35 ± 12.5 [20–58]	64	
History of perio	none	1	1	
REC baseline (mm)	4.0 ± 1.41 [2–8]	5.0 ± 1.98 [3–11]	6.0	
Maxillary rec	9	4		
Mandibular rec	11	20	2	
*Data at examination*				
PPD	1.4 ± 0.51 [1, 2]	2.0 ± 1.5 [1–7]	2	
*BOP (on buccal surfaces)*				
YES	*n* = 6	*n* = 9	*n* = 0	
NO	*n* = 14	*n* = 15	*n* = 0	
*Pl*				
YES	*n* = 8	*n* = 16	*n* = 0	
NO	*n* = 12	*n* = 8	*n* = 2	
CAL	2.6 ± 1.12 [1–5]	4.4 ± 1.82 [2–9]	5 [0]	
*FMPS*				
< 10%	95%	58.3%	100%	
10–20%		41.7%		
> 20%	5%			
RecRed (mm)	2.85 ± 1.79 [1–8]	2.67 ± 2.14 [0–9]	3.0	0.595
RC (%)	69.1 ± 23.6* [20–100]	49.2 ± 26.7* [0–83]	50%	0.016*
KTW (mm)	4.2 ± 2.0 [1–7]	3.5 ± 1.93 [1–8]	6.5 ± 0.7 [6, 7]	
RES by examiner	5.15 ± 1.74* [2–8]	3.93 ± 1.98* [1–7]	3	0.011*

*REC*, Recession; *PPD*, Probing pocket depth; *BoP*, Bleeding on probing; *Pl*, Plaque score at the treated site; *CAL*, Clinical attachment level; *FMPS*, Full mouth plaque score; *RecRed*, Recession reduction; *RC*, Root coverage; *KTW*, Keratinized tissue width; *Q-3*, Question 3; *RES*, Root coverage esthetic score.

*Significantly higher for RT1 than RT2

A mean RC of 51.9% was achieved for the mandible and 46.1% for the maxilla. A total of 10.9% of the defects showed complete RC, and 6.5% no RC improvement. Of the RT1 defects 75% resulted in ≥ 60% RC, as compared to 50% of the RT2-defects. The mean keratinized tissue width was 3.96 mm postoperatively. Of all patients, 29 were treated by postgraduate students in periodontology under supervision and 5 patients were treated by academic staff.

### Patient-reported outcomes

**Fig. 2:**
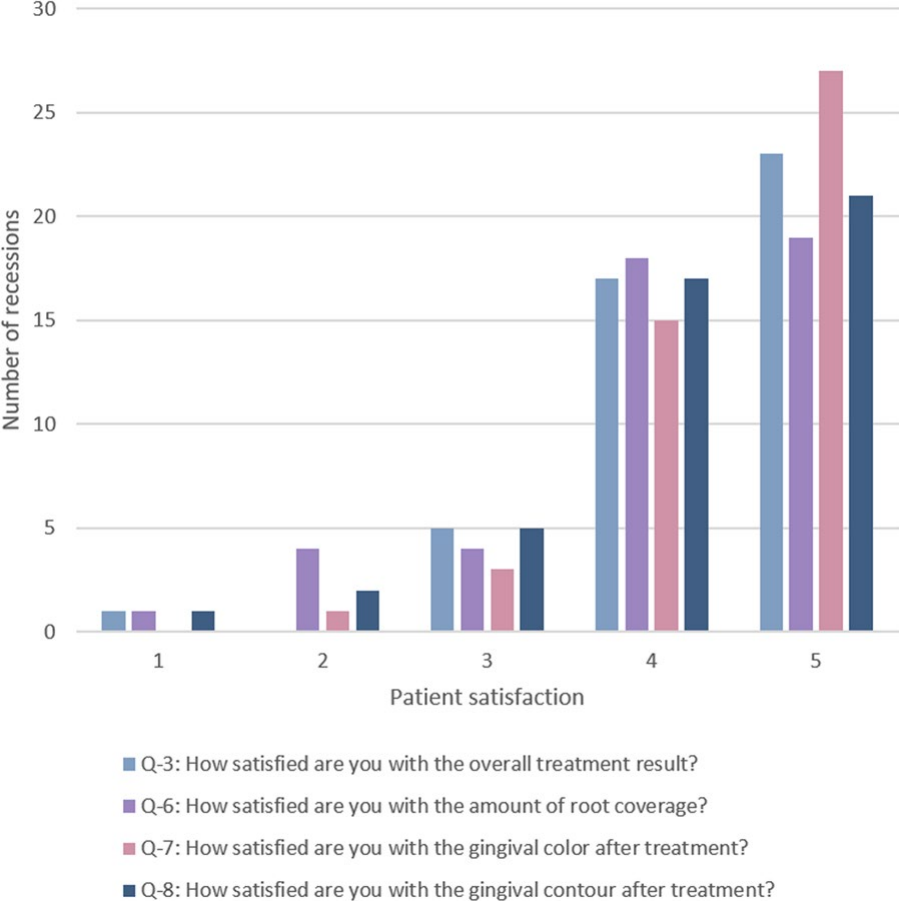
Patient-reported satisfaction

**Fig. 3:**
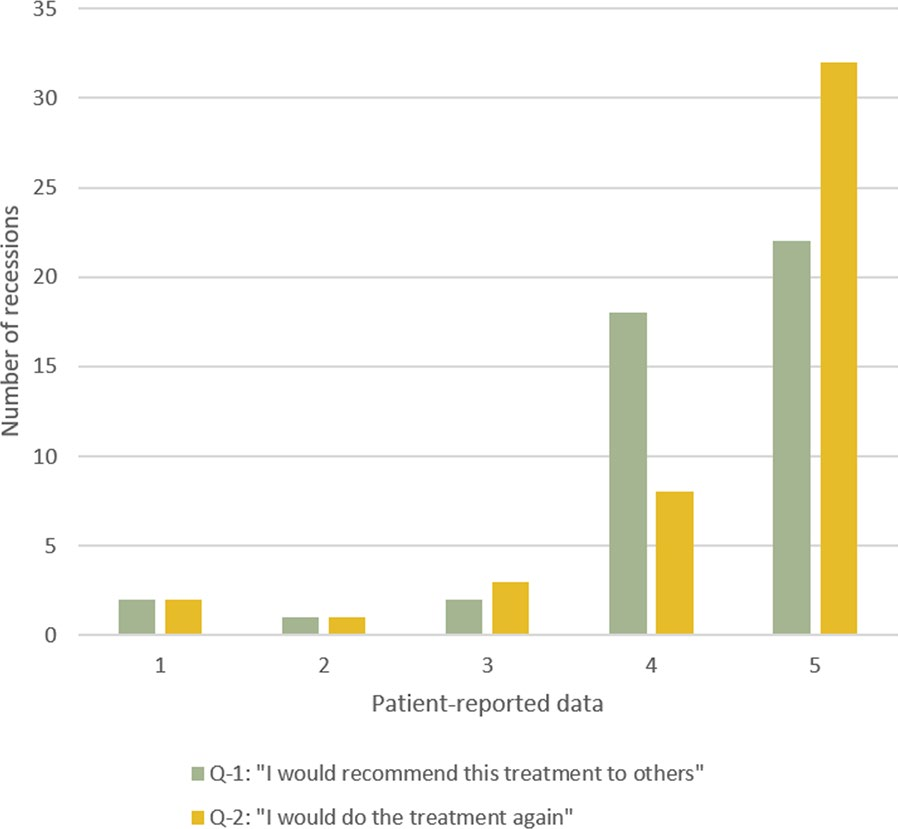
Patient-reported data

The majority of patients were satisfied (4) or very satisfied (5) with the overall treatment outcome, RC, GC and gingival contour (Fig. 2), and in retrospect most of the treated patients would have undergone the treatment again (Fig. 3).

**Fig. 4:**
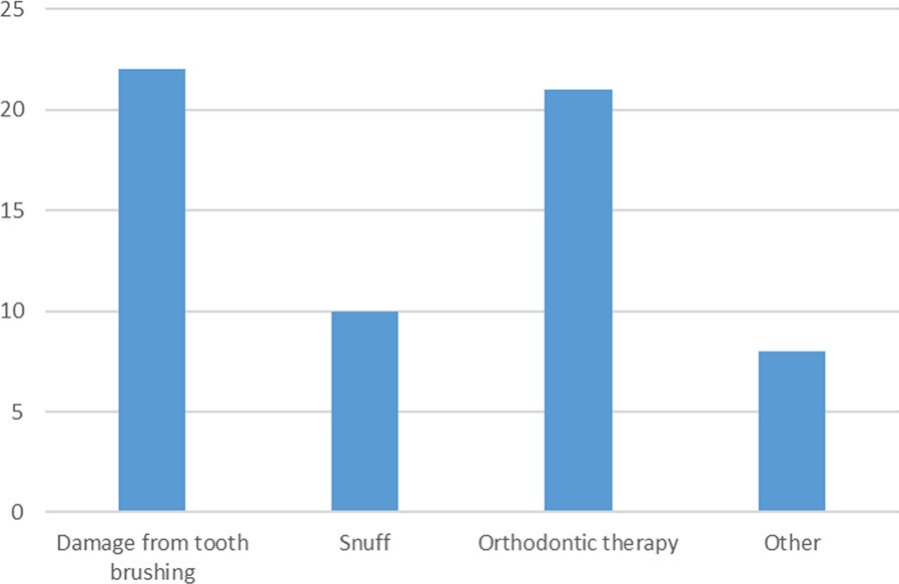
Patient-reported etiology

**Fig. 5:**
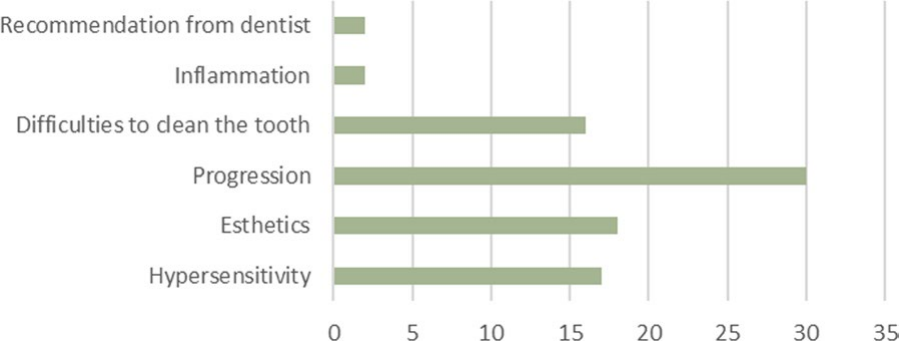
Patient-reported indication for treatment

Patient-reported reasons for seeking treatment and etiology are listed in Figs. 4 and 5. Twelve patients reported only a single reason for 17 teeth, whereas for 29 teeth 22 patients reported multiple reasons for seeking treatment.

Among the recessions presenting hypersensitivity prior to treatment, 70% reported improvement after treatment, 25% reported uncertain improvement and 5% reported no improvement.

Of the treated patients, 65% had received orthodontic treatment earlier in life, and among these 68% reported orthodontic treatment as possible etiology for the gingival recession defects. Among the patients that had undergone previous orthodontic treatment, 82.1% of the treated recessions were in the mandible. In 5 of the treated recessions, orthodontic treatment was performed in conjunction with the PPS. Of the patients, 67.4% reported that the relevant site was easier to clean after treatment, whereas 13.1% reported slightly easier, and 19.6% reported no difference.

### Professional assessment

All sites were included in the analysis. Table 2 shows the interrater reliability between RES by patient and RES by the dentist for treated RT1 and RT2. The calculated ICC has excellent agreement between the examiner and the patients for total RES and total mRES. When individual RES parameters were analyzed, the GC for RT1 recessions were not in agreement between the patients and examiner. For both RT1 and RT2 the ICC was higher when modified GM was considered. This applied to total mRES and total RES as well, with higher agreement by the use of mRES. Sub-analysis of total RES for teeth treated in the maxilla demonstrated a higher score than in the mandible. Only one patient exhibited RT3 recessions. It was 100% agreement between this patient and the examiner assessing RES and mRES, also for the individual parameters. These recessions were not presented. Another sub-analysis was done comparing multiple and single sites, and no statistical significant different assessment was found between the patients and the examiner.

**Table 2:** Descriptive statistics with intraclass correlation coefficient (ICC) for evaluation of RES and mRES by patient and examiner for RT1 and RT2

	RT1				RT2			
	Examiner median (IQR)	Patient median (IQR)	ICC (95% CI)	*p* value	Examiner median (IQR)	Patient median (IQR)	ICC (95% CI)	*p* value
TOT RES (0–10)	6.0 (2.0)	5.5 (3.75)	0.879 (0.65–0.95)	< 0.001	4.0 (2.0)	4.0 (1.75)	0.774 (0.40–0.91)	< 0.001
GM (0,3,6)	3.0 (2.25)	3.0 (3.0)	0.955 (0.88–0.98)	< 0.001	3.0 (0.0)	3.0 (0.0)	0.676 (0.27–0.86)	0.004
mGM (0–6)	5.0 (3.75)	5.0 (3.75)	0.983 (0.95–0.99)	< 0.001	4.0 (1.75)	3.5 (2.0)	0.884 (0.74–0.95)	< 0.001
MTC (0,1)	0.0 (1.0)	1.0 (0.75)	0.612 (0.05–0.84)	0.008	0.0 (1.0)	1.0 (1.0)	0.864 (0.68–0.94)	< 0.001
STT (0,1)	0.0 (1.0)	0.0 (1.0)	0.732 (0.34–0.89)	0.003	0.0 (0.0)	0.0 (0.0)	0.930 (0.84–0.97)	< 0.001
MGJ (0,1)	1.0 (1.0)	1.0 (0.75)	0.743 (0.35–0.89)	0.001	0.0 (0.75)	0.0 (1.0)	0.819 (0.58–0.92)	< 0.001
GC (0,1)	0.0 (1.0)	0.0 (1.0)	− 0.203 (− 2.23–0.53)	0.649	0.0 (1.0)	1.0 (1.0)	0.614 (0.15–0.83)	0.009
TOT mRES (0–10)	6.0 (3.75)	7.0 (4.75)	0.928 (0.78–0.97)	< 0.001	4.0 (2.0)	5.0 (2.0)	0.843 (0.59–0.94)	< 0.001

*RES*, root coverage esthetic score; *mRES*, modified RES (0–6); *TOT*, total; *GM*, gingival margin; *mGM*, modified gingival margin; *MTC*, marginal tissue contour; *STT*, soft tissue texture; *MGJ*, mucogingival junction alignment; *GC*, gingival color; *IQR*, interquartile range

Only one recession site received the highest RES score of 10, from both the patient and the examiner.

A significantly higher RES was observed for TUN + CTG as compared to VES ± CTG (*p* = 0.01). For mRES, TUN + CTG was higher than both VES ± CTG and CAF + CTG (*p* = 0.01 and *p* = 0.04, respectively).

A weak correlation (correlation coefficient (r) = 0.29, *p* = 0.054) between patient-assessed RES and PRO (Q-3) was observed, and significantly moderate correlation by using total mRES and PROs (r = 0.36, *p* = 0.017). There was a moderate correlation (r = 0.39, *p* = 0.007) between keratinized tissue width and patients finding it easier to keep it clean around the treated teeth. Table 3 describes the relation between different levels of recession type, RC, RES and patient satisfaction with statistically significant differences between each group.

**Table 3:** Assessment according to various esthetic measurements

	Number (*n*) RT1	Root coverage (%) RT1	Number (*n*) RT2	Root coverage (%) RT2	RES by examiner	RES by patient	Patient satisfaction (1–5)
Root coverage (%)							
< 50	2	22.5	8	16.4	3.6^	4.3^	3.7
50–99	13	64.4	16	65.8	4.3*	4.8*	4.5
100	5	100			7.2*^	8.4*^	4.8
*p*- value					< 0.004	0.002	
RES by examiner							
< 4	4	50.5	9	42.9	2.6*^	3.7*^	4.3
4–5	5	66.8	13	53.5	4.4*`	5.1*`	4.1
6–7	10	74.6	2	50	6.3^`	6.9^`	4.5
≥ 8	1	100			9	10	5
*p*-value					< 0.001	< 0.003	
RES by patient							
< 4	1	67	3	44.7	2.25*^`	2.25*^`	4
4–5	9	57.7	15	48.2	3.9*	4.3*”}	4.2
6–7	5	53.8	6	54.2	5.5^	6.5^”	4.5
≥ 8	4	91.8			6.5`	8.5`}	4.75
*p*-value					< 0.005	< 0.004	
Patient satisfaction							
≤ 2 poor to fair	1	67			5	5	1
3 neutral	1	20	4	11.25	3.4	4.2	3
≥ good to excellent	18	71.9	20	56.9	4.66	5.34	4.5

Patient satisfaction = 1: poor; 2: fair; 3: neutral; 4: good; 5: excellent.

*RES*, root coverage esthetic score;

^*^^`}Statistically significant difference between each group using the same esthetic measurement (*p* < *0.05*)

## Discussion

The present study was designed to compare RES by the patient to that of a dental professional. No statistically significant difference was found comparing total RES or mRES, although patients in general deemed outcomes as better than the professional. In general, the studied population reported to be satisfied with the treatment outcome. Even when patients scored low in RES, satisfaction seemed to be high. The similar RES outcomes between patients and the professional suggests that patients managed to understand and apply the RES-system.

This study is the first to compare patient-evaluated RES to that of a professional. The RES is largely based on the level of the GM following treatment which accounts for 6 out of a total of 10 points. The original RES has only a single score for partial RC irrespective of it being 10%, 50% or 90%. By modifying this parameter to have five different scores for partial RC 1–5, we hypothesized that this would reflect a higher disagreement between the patient and professional assessment, but no statistically significant difference between the two assessments of mRES was found. Interestingly, the agreement was higher when conducting mRES.

**Fig. 6:**
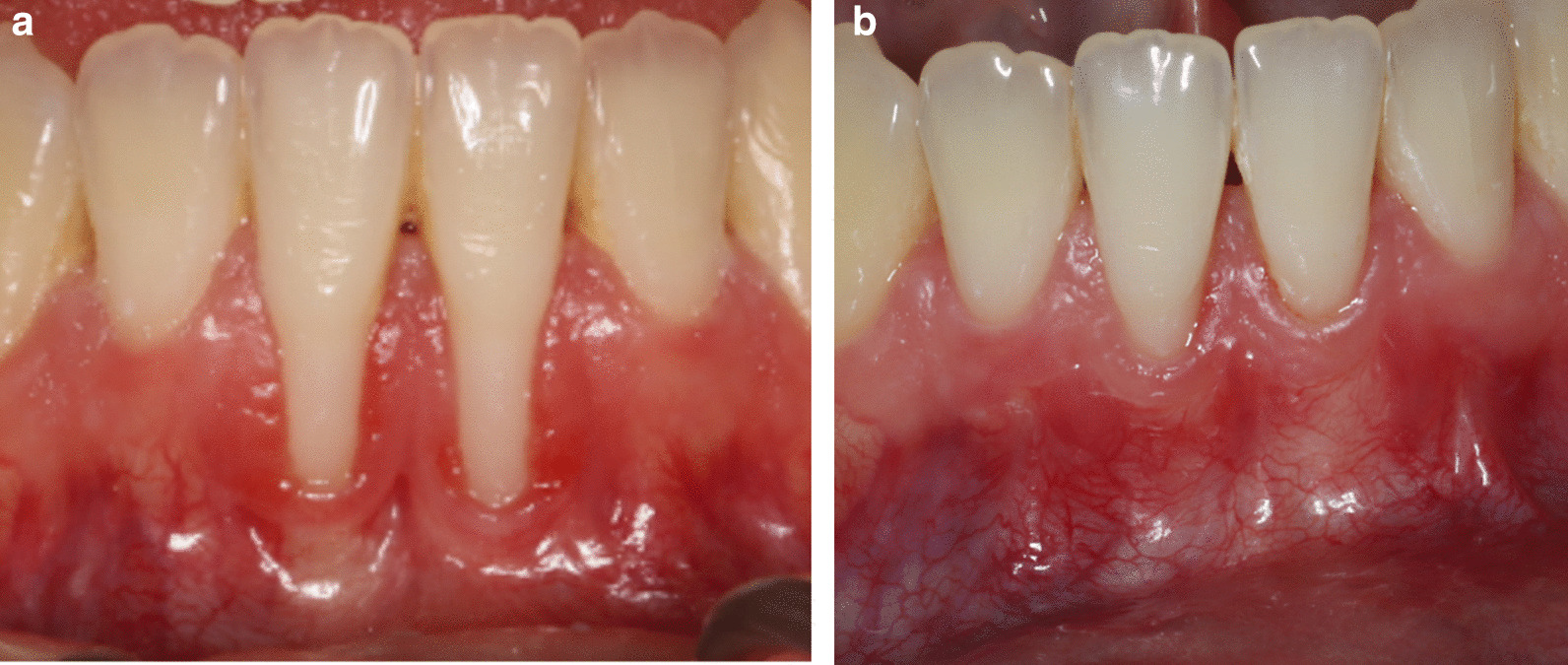
**A** Pre-operative photo, RT2. **B** 13 months post-operatively after CAF + CTG. RES by patient was scored to 6, and mRES by patient was scored to 8 for tooth #31. RES by examiner was 5, and mRES by examiner was 7 for tooth #31. For tooth #41 RES by patient was 7, and mRES was 8. Patient satisfaction scored 5 on both teeth

**Fig. 7:**
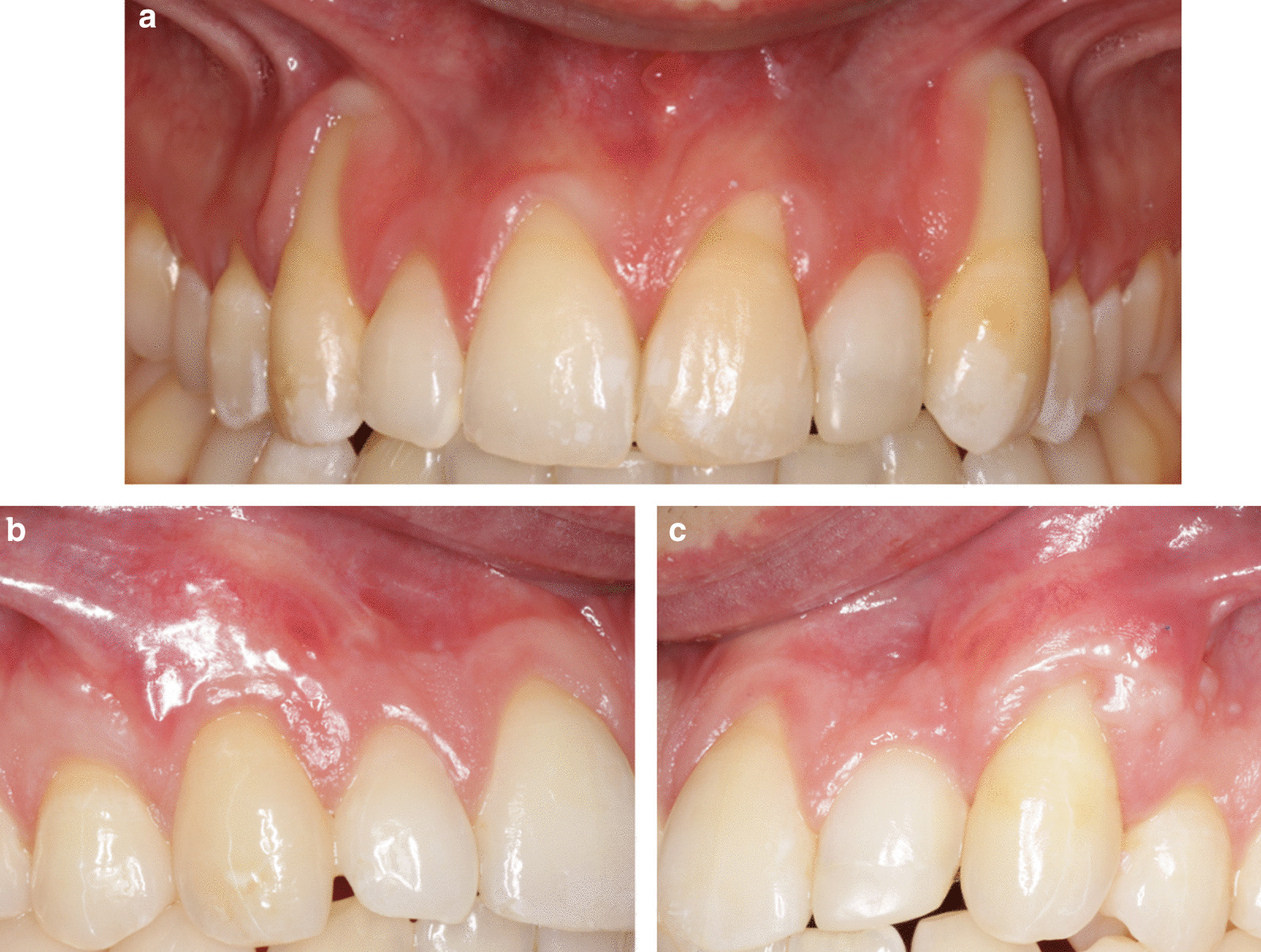
**A** Pre-operative, tooth #13: RT1, tooth #23: RT2. **B** Tooth #13 7 months postoperatively. after CAF + CTG. RES by patient was 9, mRES was 9, RES by examiner was 7 and mRES was 7. Patient satisfaction score was 5. **C** Tooth #23 6 months postoperative after two surgeries, first CAF + CTG, then CAF only. RES by patient was 4 and mRES was 6, RES by examiner was 3 and mRES was 5. Patient satisfaction score was 5

**Fig. 8:**
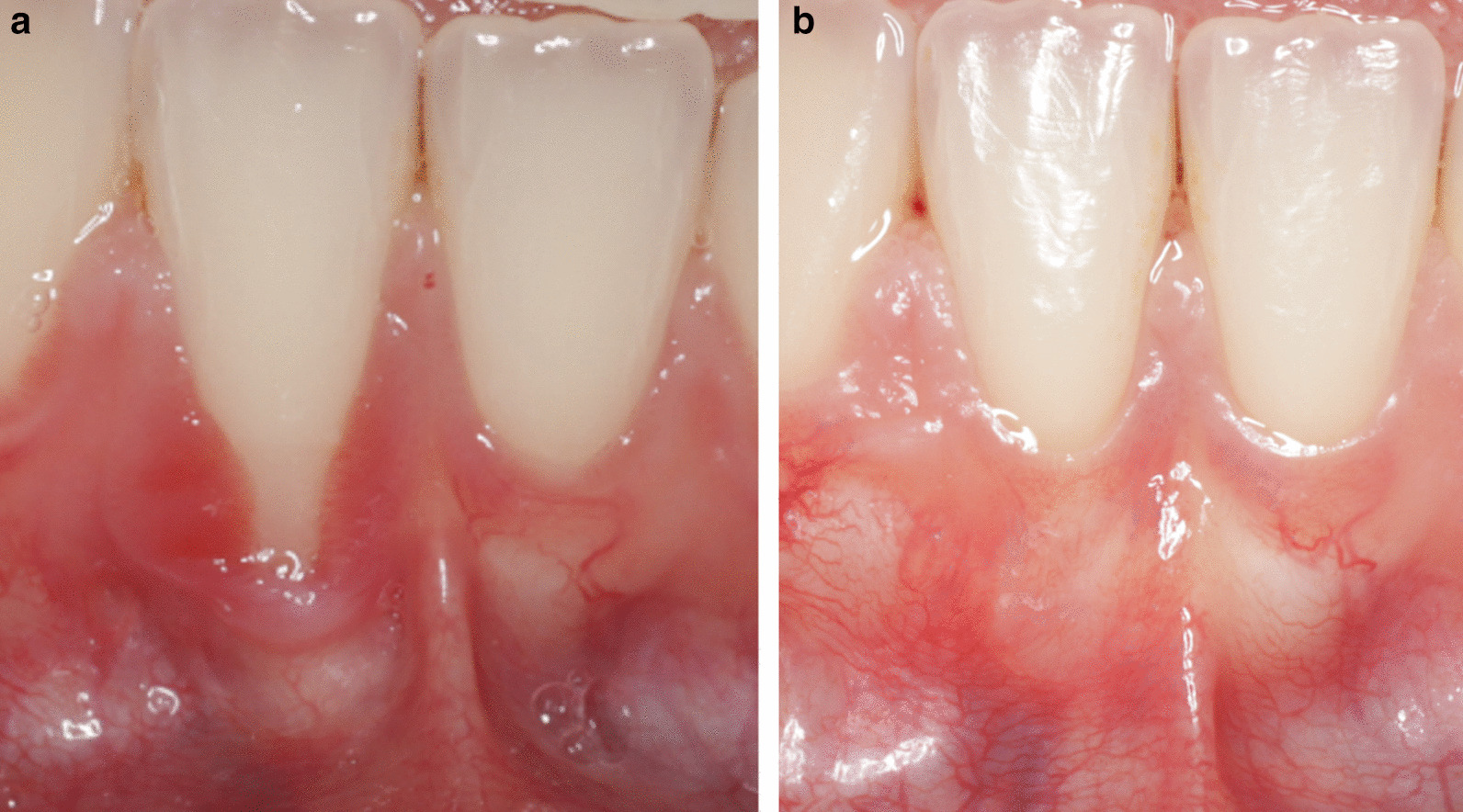
**A** Pre-operatively, RT1. **B** 21 months post-operative after CAF + CTG. RES by patient was 8 and mRES was 8 for tooth #41. RES by examiner was 7, and mRES was 7. Patient satisfaction score 4

**Fig. 9:**
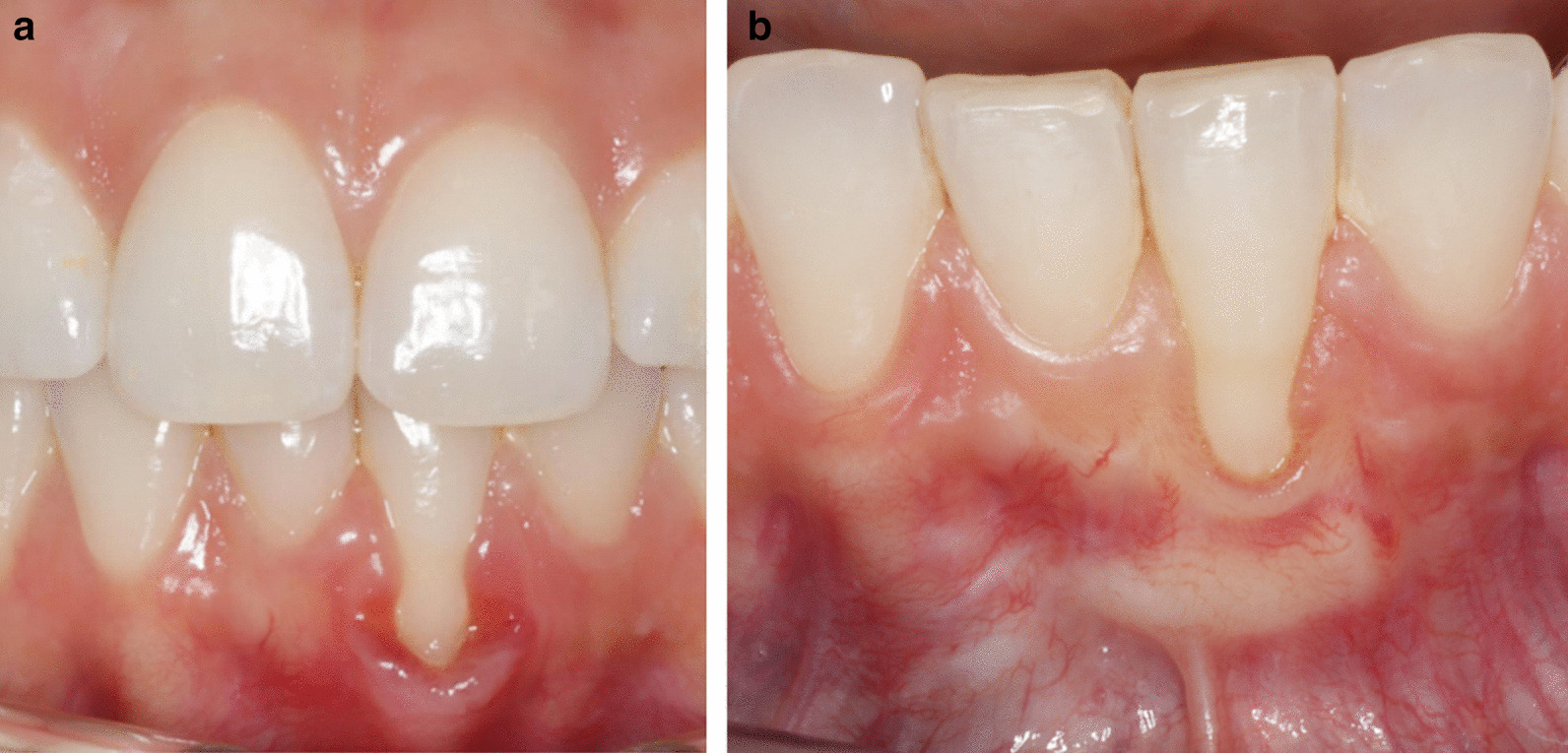
**A** Pre-operatively, RT1. **B** 27 months post-operative after CAF + CTG. RES by patient was 4, mRES was 3, RES by examiner was 3 and mRES was 1. Patient satisfaction score was 4

**Fig. 10:**
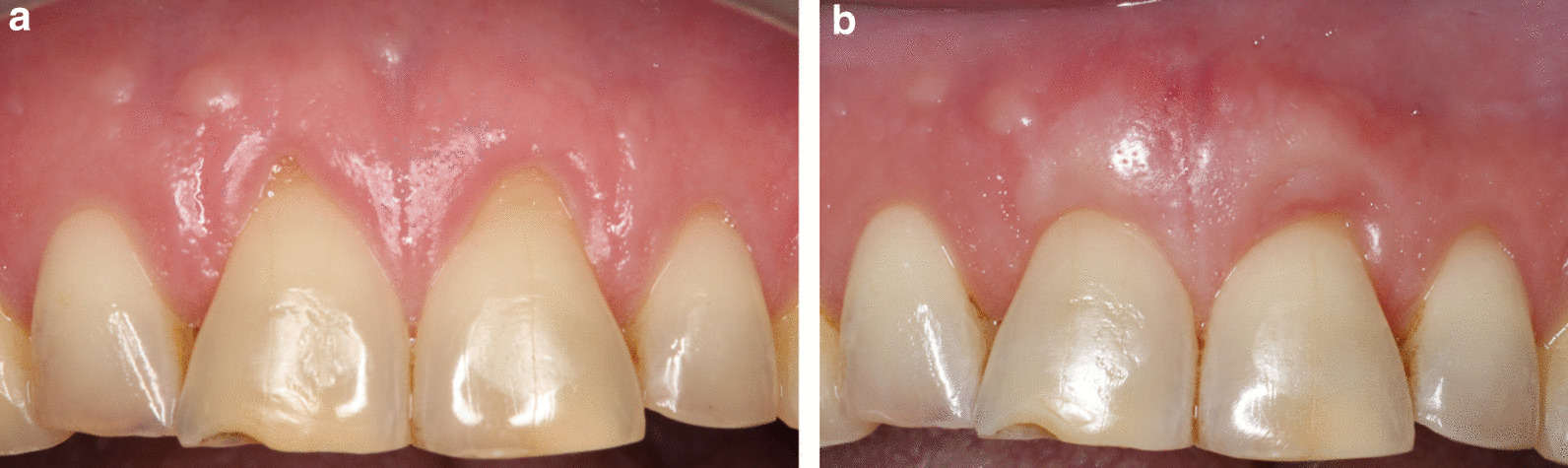
**A**: Pre-operative, RT1 for both #11 and tooth #21. **B**: 6 months post-operative after TUN + CTG. For tooth #11 RES by patient was 10, and mRES was 10. RES by examiner was 8 and mRES was 8. For tooth #21 RES by patient was 9 and mRES was 9, RES by examiner was 5 and mRES was 6. Patient satisfaction score was 5 for both teeth

Although RES factors are readily identified by clinicians, little is known about how they are rated by patients [28]. GC was the only parameter not in agreement between patient and examiner in this study (Table 2). This may be due to difficulty for the patient to distinguish keratinized and scar tissue, or the shading of the tissues. In the case of GC, the examiner evaluated this parameter more strictly. RES is a reliable method for assessing the esthetic outcomes of RC procedures among experienced periodontists [29], and a “moderately” reliable scoring system among operators with different levels of experience [30]. We therefore wanted the patient to evaluate the same factors as the clinician to better relate the clinical outcome to patient satisfaction. Few patients were unsatisfied, which implies that complete RC may not be pivotal for satisfaction for this group of patients (Figs. 6, 7, 8, 9, 10).

A few studies have assessed both professional evaluation by RES, and patient evaluation, by questionnaires based on Likert scale or visual analogue scale (VAS) [17, 19–22, 31]. The present study found a strong correlation between total subjective RES and PRO (Q-3) and even stronger with modified RES. These findings are in accordance with a previous study reporting PROs after RC procedures [19]. The authors found patient satisfaction in proportion with RES and found different perceptions regarding the importance of RC between clinicians and patients, where the clinicians considered RC as more important for a successful outcome [19]. This is also in line with the present study where the trend is high patient satisfaction despite partial RC and low RES. Partial RC may be considered a positive outcome in cases of deep gingival recession defects [32]. Another previous study found a significant positive correlation comparing PRO and RES [17], but conflicting results has also been reported [31].

A sub-analysis demonstrated higher RES/mRES following the use of a tunneling procedure with the use of a CTG as compared to CAF + CTG and VES ± CTG. Importantly, very few TUN and VES procedures were included as compared to CAF, and the study was not designed to address RES according to treatment procedure.

The mean RC achieved for RT1 was 69.1%, for RT2 49.2% and RT3 50%. There are various RC outcomes in the literature [16], and several factors predicting the outcomes of RT1 and RT2 [33]. Some studies have highlighted the fact that full RC cannot always be expected and achieved [33–35]. One of these studies used a method to predetermine the maximum RC achievable. They found that in the cases with underestimated level of RC, this was consistent with both clinical and esthetic success [34]. The patients included in the present study were individually informed of realistic clinical outcomes depending on the RT. This may have reduced patient expectations for esthetic outcome and final RC, and thereby the high patient satisfaction.

The present study has several limitations. The recessions were heterogenic due to severity and location. The surgeries were performed by clinicians with different training in the field, of varying complexity and different surgical procedures were used. In addition, the majority of recessions were located in the mandible, where esthetic demands may not be as high as in the maxilla. Another limitation may be the use of a mirror for patient RES evaluation. The use of post-operative photographs would render better details than a mirror. However, this would require standardized image acquisition, which was not feasible in the present study design. Also, for an equitable RES evaluation, the dentist would then also have to consider photographs, which may limit the clinical RES assessment. RES was conducted by the patient prior to the questionnaire, and this could have had an impact on the questionnaire answers. In the present study 10 of the identified potential 44 subjects were not willing to participate. It is not known whether their satisfaction is in line with the 34 subjects included. However, since both operators and patients were numerous, one may argue that the results may apply to everyday clinical practice. The results from this study may guide clinicians to better understand patients’ end point of therapy. In future studies RES by patients may be implemented when comparing clinical results with PROs.

## Conclusion

Within the limitation of the present study, RES evaluation by patients was not statistically significantly different to that of the professional. Patient satisfaction did not always reflect RES and clinical outcomes. Overall, patients were satisfied with the treatment result, despite partial RC. Patient satisfaction did not depend on complete RC or other parameters in RES.

## Data Availability

The dataset is not publicly available due to general data protection regulations but is available from the corresponding author on reasonable request.

## Abbreviations

PPS: Periodontal plastic surgery
RES: Root coverage esthetic score
mRES: Modified root coverage esthetic score
PRO: Patient-reported outcome
CEJ: Cemento-enamel junction
CTG: Connective tissue graft
RC: Root coverage
RT: Recession type
GM: Gingival margin
MTC: Marginal tissue contour
STT: Soft tissue texture
GC: Gingival color
MGJ: Mucogingival junction
PPD: Periodontal pocket depth
CAL: Clinical attachment level
BoP: Bleeding on probing
FMPS: Full mouth plaque score

## References

[1] Lindhe JL (2015). NP: clinical periodontology and implant. Dentistry.

[2] Tonetti MS, Jepsen S (2014). Clinical efficacy of periodontal plastic surgery procedures: consensus report of Group 2 of the 10th European workshop on periodontology. J Clin Periodontol.

[3] Kassab MM, Cohen RE (2003). The etiology and prevalence of gingival recession. J Am Dent Assoc.

[4] West NX, Sanz M, Lussi A, Bartlett D, Bouchard P, Bourgeois D (2013). Prevalence of dentine hypersensitivity and study of associated factors: a European population-based cross-sectional study. J Dent.

[5] Romandini M, Soldini MC, Montero E, Sanz M (2020). Epidemiology of mid-buccal gingival recessions in NHANES according to the 2018 World workshop classification system. J Clin Periodontol.

[6] Fragkioudakis I, Tassou D, Sideri M, Vouros I (2021). Prevalance and clinical characteristics of gingival recession in Greek young adults: a cross-sectional study. Clin Exp Dent Res.

[7] Nieri M, Pini Prato GP, Giani M, Magnani N, Pagliaro U, Rotundo R (2013). Patient perceptions of buccal gingival recessions and requests for treatment. J Clin Periodontol.

[8] Miller PD (1985). A classification of marginal tissue recession. Int J Periodontics Restor Dent.

[9] Cairo F, Nieri M, Cincinelli S, Mervelt J, Pagliaro U (2011). The interproximal clinical attachment level to classify gingival recessions and predict root coverage outcomes: an explorative and reliability study. J Clin Periodontol.

[10] Chambrone L, Tatakis DN (2016). Long-term outcomes of untreated buccal gingival recessions: a systematic review and meta-analysis. J Periodontol.

[11] Rios FS, Costa RSA, Wagner TP, Christofoli BR, Goergen J, Izquierdo C, Jardim JJ, Maltz M, Haas AN (2021). Incidence and progression of gingival recession over 4 years: a population-based longitudinal study. J Clin Periodontol.

[12] Holland GR, Narhi MN, Addy M, Gangarosa L, Orchardson R (1997). Guidelines for the design and conduct of clinical trials on dentine hypersensitivity. J Clin Periodontol.

[13] De Douglas ODW, Oliveira-Ferreira F, Flecha OD, Gonçalves PF (2013). Is surgical root coverage effective for the treatment of cervical dentin hypersensitivity? a systematic review. J Periodontol.

[14] Mounssif I, Stefanini M, Mazzotti C, Marzadori M, Sangiorgi M, Zucchelli G (2018). Esthetic evaluation and patient-centered outcomes in root-coverage procedures. Periodontol 2000.

[15] Cairo F, Rotundo R, Miller PD, Pini Prato GP (2009). Root coverage esthetic score: a system to evaluate the esthetic outcome of the treatment of gingival recession through evaluation of clinical cases. J Periodontol.

[16] Zucchelli G, Mounssif I (2015). Periodontal plastic surgery. Periodontol 2000.

[17] Stefanini M, Jepsen K, de Sanctis M, Baldini N, Greven B, Heinz B, Wennström J, Cassel B, Vignoletti F, Sanz M (2016). Patient-reported outcomes and aesthetic evaluation of root coverage procedures: a 12-month follow-up of a randomized controlled clinical trial. J Clin Periodontol.

[18] Zucchelli G, Stefanini M, Ganz S, Mazzotti C, Mounssif I, Marzadori M (2016). Coronally advanced flap with different designs in the treatment of gingival recession: a comparative controlled randomized clinical trial. Int J Periodontics Restor Dent.

[19] Kim SM, Choi YH, Kim YG, Park JW, Lee JM, Suh JY (2014). Analysis of the esthetic outcome after root coverage procedures using a comprehensive approach. J Esthetic Restor Dent Off Publ Am Acad Esthetic Dent.

[20] McGuire MK, Scheyer ET (2010). Xenogeneic collagen matrix with coronally advanced flap compared to connective tissue with coronally advanced flap for the treatment of dehiscence-type recession defects. J Periodontol.

[21] McGuire MK, Scheyer ET (2016). Long-term results comparing xenogeneic collagen matrix and autogenous connective tissue grafts with coronally advanced flaps for treatment of dehiscence-type recession defects. J Periodontol.

[22] Zucchelli G, Mounssif I, Mazzotti C, Montebugnoli L, Sangiorgi M, Mele M, Stefanini M (2014). Does the dimension of the graft influence patient morbidity and root coverage outcomes? a randomized controlled clinical trial. J Clin Periodontol.

[23] O'Leary TJ, Drake RB, Naylor JE (1972). The plaque control record. J Periodontol.

[24] Nyman S, Lindhe J, Lundgren D (1975). The role of occlusion for the stability of fixed bridges in patients with reduced periodontal tissue support. J Clin Periodontol.

[25] Chapple ILC, Mealey BL, Van Dyke TE, Bartold PM, Dommisch H, Eickholz P, Geisinger ML, Genco RJ, Glogauer M, Goldstein M (2018). Periodontal health and gingival diseases and conditions on an intact and a reduced periodontium: consensus report of workgroup 1 of the 2017 world workshop on the classification of periodontal and peri-implant diseases and conditions. J Periodontol.

[26] de Sanctis M, Zucchelli G (2007). Coronally advanced flap: a modified surgical approach for isolated recession-type defects: three-year results. J Clin Periodontol.

[27] Zuhr O, Fickl S, Wachtel H, Bolz W, Hürzeler MB (2007). Covering of gingival recessions with a modified microsurgical tunnel technique: case report. Int J Periodontics Restor Dent.

[28] Cortellini P, Bissada NF (2018). Mucogingival conditions in the natural dentition: narrative review, case definitions, and diagnostic considerations. J Periodontol.

[29] Cairo F, Nieri M, Cattabriga M, Cortellini P, De Paoli S, De Sanctis M, Fonzar A, Francetti L, Merli M, Rasperini G (2010). Root coverage esthetic score after treatment of gingival recession: an interrater agreement multicenter study. J Periodontol.

[30] Isaia F, Gyurko R, Roomian TC, Hawley CE (2018). The root coverage esthetic score: intra-examiner reliability among dental students and dental faculty. J Periodontol.

[31] Zuhr O, Rebele SF, Schneider D, Jung RE, Hürzeler MB (2014). Tunnel technique with connective tissue graft versus coronally advanced flap with enamel matrix derivative for root coverage: a RCT using 3D digital measuring methods. Part I. Clinical and patient-centred outcomes. J Clin Periodontol.

[32] Rotundo R, Nieri M, Mori M, Clauser C, Prato GP (2008). Aesthetic perception after root coverage procedure. J Clin Periodontol.

[33] Ozcelik O, Seydaoglu G, Haytac MC (2015). Prediction of root coverage for single recessions in anterior teeth: a 6-month study. J Clin Periodontol.

[34] Zucchelli G, Mele M, Stefanini M, Mazzotti C, Mounssif I, Marzadori M, Montebugnoli L (2010). Predetermination of root coverage. J Periodontol.

[35] Aichelmann-Reidy ME, Yukna RA, Evans GH, Nasr HF, Mayer ET (2001). Clinical evaluation of acellular allograft dermis for the treatment of human gingival recession. J Periodontol.

